# Comparison Between Mini-Sternotomy and Full Sternotomy for Aortic Valve Replacement: A 10-Year Retrospective Study

**DOI:** 10.7759/cureus.31627

**Published:** 2022-11-17

**Authors:** Ahmad Faraz, Nerielle Fundano, Ammal Imran Qureshi, Muhammad Yasir Tarar, Bakhat Yawar, Ghulam Dastagir Faisal Mohammed

**Affiliations:** 1 General Surgery, Ulster Hospital, Dundonald, GBR; 2 Cardiothoracic Surgery, Royal Victoria Hospital, Belfast, GBR; 3 Surgery, Worthing Hospital, Southampton, GBR; 4 Trauma and Orthopaedics, Salford Royal NHS Foundation Trust, Manchester, GBR; 5 General Surgery, The Western Trust Health and Social Care (Northern Ireland), Derry/Londonderry, GBR; 6 Neurological Surgery, Salford Royal NHS Foundation Trust, Manchester, GBR

**Keywords:** length of hospital stay, retrospective study, full sternotomy, ministernotomy, aortic valve replacement

## Abstract

Introduction

Aortic valve replacement (AVR) is a mainstay treatment for moderate to severe aortic valve stenosis. This retrospective study aimed to compare the clinical outcomes of mini-sternotomy and conventional sternotomy.

Methodology

This 10-year retrospective study compared the clinical outcomes of mini-sternotomy and full sternotomy. Patient-related outcomes include sternal wound dehiscence, operative time, length of hospital stay, and Intensive Care Unit (ICU) stay, whereas intraoperative parameters such as cardiopulmonary bypass (CPB) time and Aortic Cross Clamp time (ACCt) were compared between the two treatment groups.

Results

A total of 371 patients underwent AVR. Among them, 238 patients had AVR with full sternotomy and 133 patients had a mini-sternotomy. Full sternotomy patients had significantly lower bleeding than those in the mini-AVR group (p-0.002). The operation time was also found to be significantly higher in the mini-AVR group. The duration of hospital stays, ICU stay, and deep sternal wound dehiscence were recorded to be statistically insignificant between the two treatment groups. Atrial fibrillation, sternal wound dehiscence, stroke and perioperative myocardial infarctions, were equally observed between the two groups.

Conclusion

Mini-sternotomy is a safe option for AVR. The same number of complications were observed between the two groups; however, there was a reduction in the duration of hospital stay and ICU stay amongst the mini-sternotomy group.

## Introduction

Aortic valve degeneration affects 2-7% of people aged 65 years old or above and is the fourth most prevalent cardiovascular condition in the modern world [1]. Aortic stenosis is the most prevalent defect type which is caused by valve degradation. When left untreated, this can lead to myocardial hypertrophy, prolonged left ventricular ejection time, and reduction in contractility, enlarging the left ventricle and raising end-diastolic pressure. Aortic valve replacement (AVR) is the recommended therapy for haemodynamically severe aortic valve disease even if medical treatment can assist in reducing symptoms. In the era of transcatheter aortic valve replacement, there has been an increase in interest in minimally invasive methods for open AVR [2,3]. The present study has been aimed to compare the outcomes of AVR through mini-sternotomy vs full sternotomy. The mini-sternotomy approach involves smaller skin incisions that lead to better cosmetic outcomes [4,5]. Additionally, this approach has been reported to be associated with minimal bleeding time, shorter Intensive Care Unit (ICU) and overall hospital stay.

## Materials and methods

Study design

This retrospective study was conducted at our regional cardiac surgery unit, which is a referral centre, where patients were referred from the district general hospitals for congenital and adult cardiac surgery. We collected data from patients who underwent AVR through mini-sternotomy and full sternotomy for a 10-year period, i.e., from 28 January 2012 to 27 November 2021. Data was gathered by reviewing the Electronic Care Records (ECR) of patients who underwent AVR during these 10 years. This investigation was classified as a retrospective cohort study based on the guidelines from the National Health Service Health Research Authority (NHS HRA); hence, no formal ethical approval or institutional review board approval was required. However, this study was registered at the regional quality improvement department. Patient outcomes such as blood loss (intraoperative bleed), drain output, infections, redo sternotomy and wound dehiscence, and operative time were recorded among the two groups.

Inclusion and exclusion criteria

All patients aged above 18 years old who underwent either mini-sternotomy or full sternotomy for AVR were included in this study. Patients who had chest deformities, such as scoliosis or pectus excavatum, and who underwent other cardiac surgery, such as coronary artery bypass grafting or any other valve procedure during similar surgery, and patients with infective endocarditis were excluded from the study.

Surgical technique

Six experienced cardiac surgeons performed AVR at our regional unit. Mini-sternotomy was carried out with a 6-8 cm long incision that extended from the head of the second rib to the fourth intercostal space avoiding injury to the right internal mammary artery. Thymic fat was dissected, and pericardial stay sutures were applied along with sternal retractors to improve exposure to the chest cavity. Patients were fully heparinised, followed by aortic cannulation into ascending aorta by Seldinger technique. Venous cannulation was performed via the Right atrium and inferior vena cava (IVC). Aortic cross-clamp patient goes on cardiac pulmonary bypass.

Full sternotomy was done via conventional median sternotomy incision, followed by cannulation of ascending aorta, right atrium and inferior vena cava.

The two most common types of valves used in our unit were Perimount Magna Ease (Edwards LLC, Irvine, CA) for tissue valve replacement and St. Jude Medical (St. Paul, MN) and CarboMedics (Austin, TX) mechanical heart valves.

Data analysis

Data analysis was performed using the Statistical Package for the Social Sciences (SPSS) version 25 (IBM Corp., Armonk, NY). Mean and standard deviation was used to analyse continuous variables. The analysis was completed with a student t-test. Chi-squared was employed to examine categorical variables. A p-value of less than 0.05 was considered statistically significant.

## Results

A total of 371 patients were included in the study. Among them, 238 patients underwent full median sternotomy, whereas 133 patients had mini-sternotomy for AVR. The demographic data is summarised in Table 1.

**Table 1:** Demographic Data ASA: American Society of Anesthesiologists; NYHA: New York Heart Association.

	Mini-Sternotomy (133)	Full Sternotomy (238)
Age	59.14 ± 9.76	62.34 ± 8.87
Male	82 (61.65%)	147 (61.76%)
Female	56 (42.10%)	109 (45.79%)
Body Mass Index	29.47 ± 5.4	30.18 ± 4.12
Atrial Fibrillation	57 (42.8%)	82 (34.45%)
Diabetes	22 (16.5%)	38 (15.9%)
Hypertension	48 (36.09%)	73 (30.67%)
Myocardial infarction	8 (6.01%)	31 (13.06%)
ASA 1	23 (17.29%)	49 (20.58%)
ASA II	44 (33.08%)	67 (28.15%)
ASA III	51 (38.34%)	82 (34.45%)
ASA IV	30 (22.55%)	40 (16.8%)
NYHA I	18 (13.53%)	44 (18.48%)
NYHA II	47 (35.33%)	74 (31.09%)
NYHA III	48 (36.09%)	80 (33.61%)
NYHA IV	35 (26.3%)	50 (21.00%)

In the mini-sternotomy group, 12 patients had re-sternotomy for post-operative bleeding and tamponade (Table 2). An almost similar incidence was noted amongst patients who underwent full sternotomy. We found that there was a significant difference (Table 2) in intraoperative bleeding between the two treatment groups (p-0.002). The length of ICU stay is a significant indicator of mortality and morbidity [4]. We found that the mean time for patients’ ICU stay was similar for both the mini-AVR and conventional sternotomy groups (p-0.74) as shown in Table 2. With regards to patient-related outcomes of this study, the duration of hospital stay in full sternotomy patients was longer than in mini-sternotomy patients. However, this difference was not statistically significant (p-0.345), as shown in Table 2. In our retrospective study, a significant statistical difference was found in the operative time between the two groups. The full sternotomy group had a total operative time of 191 ± 27.69, which is less than 223 ± 20.33 for the mini-sternotomy group (Table 2).

**Table 2:** Patient-Related Outcomes for Mini-Sternotomy and Full Sternotomy AVR: Aortic valve replacement

	Mini-sternotomy	Full Sternotomy AVR	p-value
Intraoperative Bleeding	247.43 (ml)	331 (ml)	p-0.002
Length of ICU Stay	3.31 ± 2.96	4.1 ± 3.61	p-0.743
Length of Hospital Stay	5.53 ± 3.22	6.67 ± 4.34	p-0.345
Operation time	223 ± 20.33	191 ± 27.69	p.031
Post-operative Bleeding	12	14	p-0.278
Redo sternotomy	12	13	p-0.333
Hemofiltration / dialysis	2	6	p-0.012
Permanent Stroke	3	1	p-0.276
Perioperative Myocardial infarction	1	0	p 0.171
Pacemaker insertion	0	3	p-0.004
Deep sternal wound infections	1	0	p- 0.321
Early Mortality	1	3	p- 0.731

Post-operative bleeding was recorded equally between the mini-AVR and conventional AVR groups (Table 2) and was not found to be statistically significant. Furthermore, they underwent re-sternotomy for the evacuation of bleeding. Compared to patients in full sternotomy, those in the mini-sternotomy had a higher number of dialysis performed postoperatively (p-0.012). Stroke, deep sternal wound infections and post-operative myocardial infarctions were recorded mostly amongst mini-AVR patients, but these results were not found to be statistically significant (Table 2). Mini-sternotomy resulted in a longer cross-clamp time compared to full sternotomy. Similarly, cardiopulmonary bypass (CPB) time was also found to be significantly lower in the conventional AVR group (Table 3).

**Table 3:** Intraoperative Parameters AVR: Aortic valve replacement

	Mini-AVR	Full Sternotomy AVR	
Cross Clamp Time (min)	84 ± 14.3	71 ± 17.4	p-0.002
Cardiopulmonary Bypass Time (min)	118 ± 20.11	96 ± 12.63	p-0.031

## Discussion

Mini-sternotomy is associated with minimal trauma, offers better stability of the sternum and thorax, provides superior aesthetic outcome and quickens the healing process with minimal sternal wound dehiscence [6]. However, mini-sternotomy's limiting view of the operative field prevents the surgeon and assistant surgeon from having an appropriate sight of the procedure and obstructs access to the ascending aorta. Additionally, it can lead to a longer procedure time. Therefore, mini-AVR is technically more challenging for a cardiac surgeon and necessitates additional skills [7].

Prolonged operation time and CPB are regarded as some of the drawbacks of the mini-AVR method. Furukawa et al. (2014) found that prolonged CPB and operative time puts patients at risk of complications. With the partial cutting of the sternum, the contractility of the myocardium and ventricular refill is challenging to assess [8]. Salis et al. (2008) recorded that prolonging CPB over 30 minutes increases the risk of postoperative mortality and morbidity [9]. Operative time and CPB are found to be longer for mini-sternotomy than for full sternotomy [9]. Hence, the present study agrees with the results of previously published data.

Mini-sternotomy is associated with minimal postoperative bleeding; it causes lesser trauma and a minimal inflammatory response [1]. Khoshbin et al. (2011) conducted a meta-analysis of randomised controlled trials (RCTs) and found no difference in blood loss between mini-sternotomy and full sternotomy [10]. However, Filip et al. (2018) noted a difference of 230 ml drainage between the two treatment groups [7]. In our study, we found a difference of 142 ml between the two groups, which was not statistically significant. Hancock et al. (2019) published an RCT of 270 patients and found that postoperative drainage in the conventional sternotomy group was higher than that in the mini-sternotomy group; this was statistically significant (p-0.0001) [11]. The present study found equal incidence of re-sternotomy secondary to bleeding or pericardial tamponade between the two treatment groups (p>0.06). Kirmani et al. (2017) published a systematic review and meta-analysis of RCTs and found that the re-exploration for bleeding was the same in mini-sternotomy and full sternotomy, i.e., the CIs for each of the studies crossed over the line of no effect, and therefore, there was no difference in the net effect between full and upper hemi‐sternotomy. They further found that deep sternal wound infections were recorded equally for these two procedures, which was not statistically significant (p>0.05) [12].

The length of hospital stay is a significant factor for predicting morbidity and mortality. The current study recorded that patients with full sternotomy had a longer duration of hospital stay than those in the mini-sternotomy group (p<0.03). Zallé et al. (2021) conducted a prospective study to compare the outcomes of full sternotomy and mini-sternotomy. The author found that the duration of hospital stay was shorter in the mini-sternotomy group than in the full sternotomy group [13]. Khoshbin et al. (2011) found that the length of ICU stay was slightly prolonged for patients who had full sternotomy, which was statistically significant (p=0.003) [10]. Our study did not find a significant difference in ICU stay between the two groups. It is worth noting that few studies had similar outcome with regard to the reduction in the duration of ICU stay amongst minimally invasive sternotomy patients [14].

Literature review reveals that mini-AVR patients have a higher rate of atrial fibrillation. Filip et al. (2018) conducted a retrospective single-centre study and observed a statistically insignificant lower rate of atrial fibrillation in mini-AVR patients. The results of our study are in line with Filip et al. (2018) [7]; however, three patients were found to have stroke.

## Conclusions

Our study confirms that mini-sternotomy is a safe option for AVR. We found that a similar number of complications were observed between the two groups. However, mini-sternotomy results in better cosmetic effect. Literature review as well as our study confirms that mini-sternotomy reduces the length of hospital and ICU stay, therefore found to be cost-effective. However, mini-sternotomy requires more surgical expertise when compared to full sternotomy. We further found that the operative time for conventional sternotomy was shorter than mini-sternotomy. Overall, a mini-sternotomy was a safe and reproducible procedure which was not associated with prolonged aortic cross-clamp time (ACCt) or cardiopulmonary bypass time (CPBt) compared with a full sternotomy, therefore, it is a valuable and safe treatment option.
